# The actin bundling activity of ITPKA mainly accounts for its migration-promoting effect in lung cancer cells

**DOI:** 10.1042/BSR20222150

**Published:** 2023-02-09

**Authors:** Lukas Küster, Themistoklis Paraschiakos, Kader Ebru Karakurt, Udo Schumacher, Björn-Philipp Diercks, Sabine Windhorst

**Affiliations:** 1 Department of Biochemistry and Signal Transduction, University Medical Center Hamburg-Eppendorf, Martinistrasse 52, D-20246 Hamburg, Germany; 2 Institue of Anatomy and Experimental Morphology, University Medical Center Hamburg-Eppendorf, Martinistrasse 52, D-20246 Hamburg, Germany; 3 Department of Biochemistry and Molecular Cell Biology, University Medical Center Hamburg-Eppendorf, Martinistrasse 52, D-20246 Hamburg, Germany

**Keywords:** actin, cell invasion, Ins(1,4,5)P3, ITPKA, metastasis, migration

## Abstract

Expression of Ins(1,4,5)P_3_-kinase-A (ITPKA), the neuronal isoform of Ins(1,4,5)P_3_-kinases, is up-regulated in many tumor types. In particular, in lung cancer cells this up-regulation is associated with bad prognosis and it has been shown that a high level of ITPKA increases migration and invasion of lung cancer cell lines. However, since ITPKA exhibits actin bundling and Ins(1,4,5)P_3_-kinase activity, it was not clear which of these activities account for ITPKA-promoted migration and invasion of cancer cells. To address this issue, we inhibited endogenous actin bundling activity of ITPKA in lung cancer H1299 cells by overexpressing the dominant negative mutant ITPKA^L34P^. Analysis of actin dynamics in filopodia as well as wound-healing migration revealed that ITPKA^L34P^ inhibited both processes. Moreover, the formation of invasive protrusions into collagen I was strongly blocked in cells overexpressing ITPKA^L34P^. Furthermore, we found that ATP stimulation slightly but significantly (by 13%) increased migration of cells overexpressing ITPKA while under basal conditions up-regulation of ITPKA had no effect. In accordance with these results, overexpression of a catalytic inactive ITPKA mutant did not affect migration, and the Ins(1,4,5)P_3_-kinase-inhibitor GNF362 reversed the stimulating effect of ITPKA overexpression on migration. In summary, we demonstrate that under basal conditions the actin bundling activity controls ITPKA-facilitated migration and invasion and in presence of ATP the Ins(1,4,5)P_3_-kinase activity slightly enhances this effect.

## Introduction

Ins(1,4,5)P_3_-kinase-A (ITPKA or InsP_3_kinase) was first described and characterized by Irvine et al. in 1986 and cloned by Takazawa et al. in 1990 [1,2]. The authors found that ITPKA phosphorylates the calcium mobilizing second messenger Ins(1,4,5)P_3_ to Ins(1,3,4,5)P_4_ and later studies revealed that Ins(1,3,4,5)P_4_ can be further phosphorylated to Ins(1,3,4,5,6)P_5_ by IPMK or dephosphorylated by a 5′phosphatase (INPP4) to Ins(1,4)P_2_. Since the affinity of the phosphatase INPP4 to Ins(1,3,4,5)P_4_ is about 10-fold higher compared with Ins(1,4,5)P_3_, an increased cellular Ins(1,3,4,5)P_4_ concentration protects Ins(1,4,5)P_3_ from dephosphorylation. Therefore, the duration and the concentration of Ins(1,4,5)P_3_-mediated calcium signals depend on the ITPKA/INPP4 ratio [3,4]. Furthermore, Ins(1,3,4,5)P_4_ itself can directly activate calcium channels and selectively binds to GAP^IP4^ (also called centaurin α1), exhibiting multiple cellular functions [3–5]. In addition to its InsP_3_-kinase activity, Johnson and Schell were the first to show that the N-terminal actin binding domain of ITPKA (ITPKA^1-66^) bundles F-actin by forming homodimers. Our group analyzed the effect of full-length ITPKA on actin dynamics and revealed an additional F-actin cross-linking activity, due to the involvement of the C-terminal InsP_3_-kinase domain in F-actin arrangement, forming tight flexible actin networks [4,6]. Moreover, the Schell group as well as our group found that a high level of ITPKA strongly alters the cellular actin cytoskeleton, in particular in cellular protrusions [4,7–9].

In normal cells the ITPKA gene expression is restricted, with the exception of mainly neurons and testis [10]. In neurons, ITPKA accumulates at dendritic spines of hippocampal, cerebral and cortical neurons, and in these cellular compartments ITPKA controls actin dynamics and calcium signaling, contributing to the regulation of synaptic plasticity [4,7,11,12]. Therefore, ITPKA knockout mice show impaired learning and memory function but no broad phenotypic limitations [11,13]. In addition to neurons, many tumor types express ITPKA, due to promoter demethylation, mutation of the transcription repressor REST, or gene body methylation [14,15]. Meanwhile, there are a lot of reports, showing that this overexpression is associated with poor clinical outcome of cancer patients, and knock-down and overexpression approaches revealed that a high level of ITPKA increases the metastatic potential of tumor cells *in vitro* and *in vivo* (reviewed in [16]). However, currently it is unknown whether the InsP_3_-kinase- or the actin bundling activity drives the metastatic potential of ITPKA.

In the present study, we addressed this issue by analyzing migration, invasion and cytoskeletal dynamics in lung cancer cells with inhibited actin bundling or InsP_3_-kinase activity. For this purpose, we overexpressed a dominant-negative ITPKA mutant blocking the actin bundling activity of ITPKA or employed a membrane-permeable InsP_3_-kinase inhibitor.

## Results

### ITPKA knock-down inhibits metastasis of lung cancer cells

**Figure 1:**
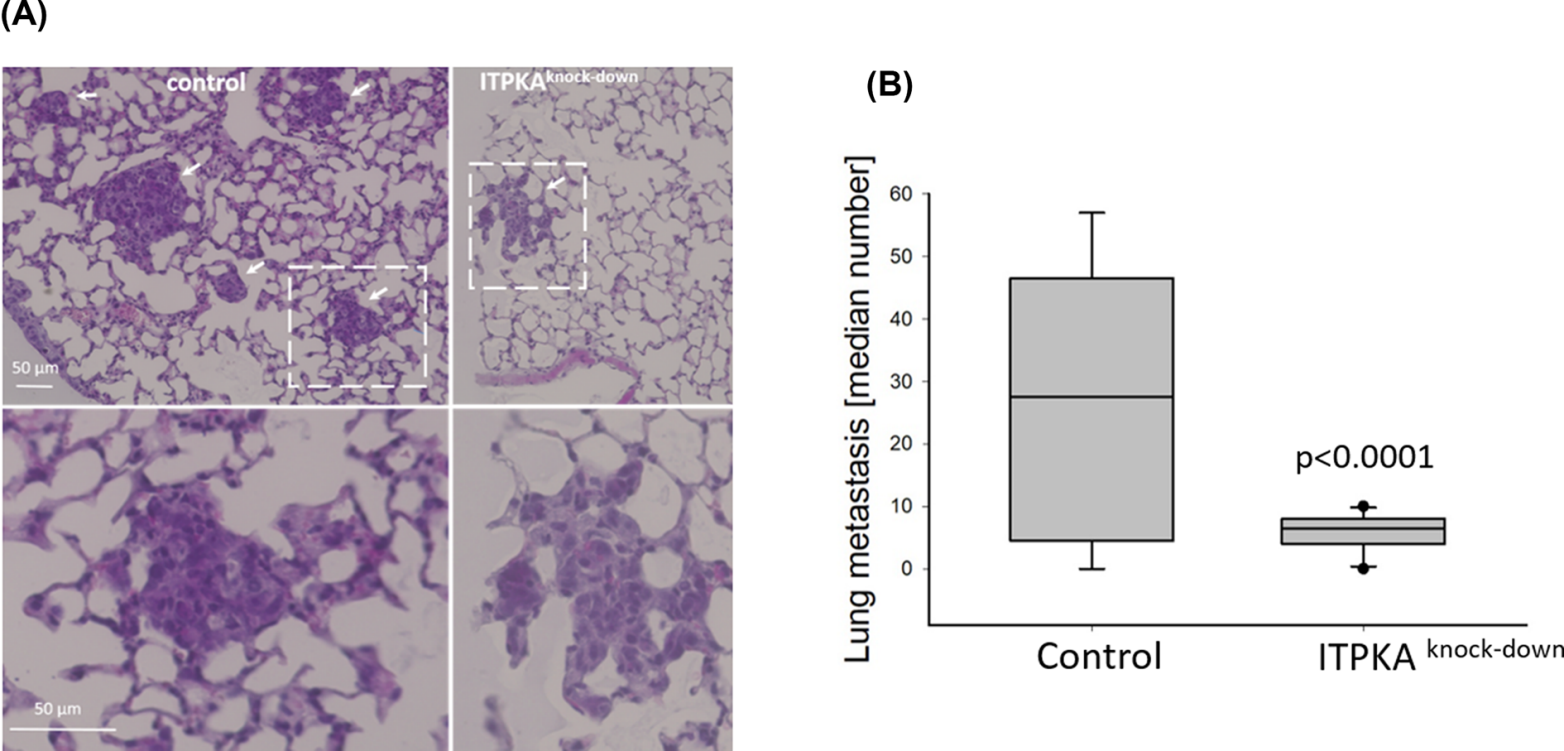
Knock-down of ITPKA strongly reduces formation of distant metastasis in mice (**A**) H1299 control and H1299 ITPKA knock-down cells [4] were subcutaneously injected into the neck of SCID mice and after visible tumors were grown, the mice were sacrificed, the lungs dissected, H&E-stained, and sliced. Representative images of lung-slices of mice inoculated with control cells (left) or with ITPKA^knock-down^ (right) cells are shown. Lung micrometastasis are marked by arrows, and amplified images in the lower panels show selected micrometastasis. (**B**) The number of lung metastases of 8 (control) or 10 (ITPKA^knock-down^) mice were determined, and median values calculated.

In a former study we revealed that down-regulation of ITPKA in lung cancer H1299 cells decreased trans-migration [4]. Since trans-migration is an important step in the metastatic cascade [17], here we analyzed the effect of ITPKA-depletion on dissemination of H1299 cells in a Xenograft-SCID mouse model. For this, control and ITPKA knock-down H1299 cells (see [4]) were subcutaneously injected into SCID mice and after 11 days, when visible tumors were grown, the mice were killed and the lungs dissected. The slices were H&E stained, and analyzed for the presence of micrometastases in the lung. In Figure 1A (right panel), representative micrographs of the H&E stained lung slices from both groups are shown. The lung metastases derived from H1299 cells are embedded in the lung tissue and marked by white arrows. In the micrograph showing the lung tissue from mice inoculated with control H1299 cells five micrometastasis are visible. In the exemplary lung tissue from mice treated with ITPKA knock-down cells only one metastasis is shown (see also magnification in the lower panel), which is embedded in normal lung tissue.

From eight mice inoculated with control cells, seven showed micrometastases in the lung, and a median of 29 metastases/lung was calculated. In nine out of ten mice treated with ITPKA-depleted cells micrometastasis in the lung were detected with a median of 6.5 (see Figure 1B). Thus, depletion of ITPKA-expression reduces dissemination of lung cancer cells *in vivo* by 78%, showing that ITPKA is essential for dissemination of H1299 lung cancer cells.

### The mutant ITPKA^L34P^ is a dominant negative mutant of actin bundling activity of ITPKA

Our data depicted in Figure 1 clearly show that ITPKA is essential for dissemination of H1299 lung cancer cells *in vivo*. However, the protein exhibits InsP_3_-kinase and F-actin bundling activity, and therefore it was not clear which activity accounts for this effect. Since the regulation of actin dynamics essentially controls metastasis of tumor cells [18], we first intended to analyze the impact of the actin bundling activity for ITPKA-controlled migration, which is one essential step in the metastatic cascade [17].

**Figure 2:**
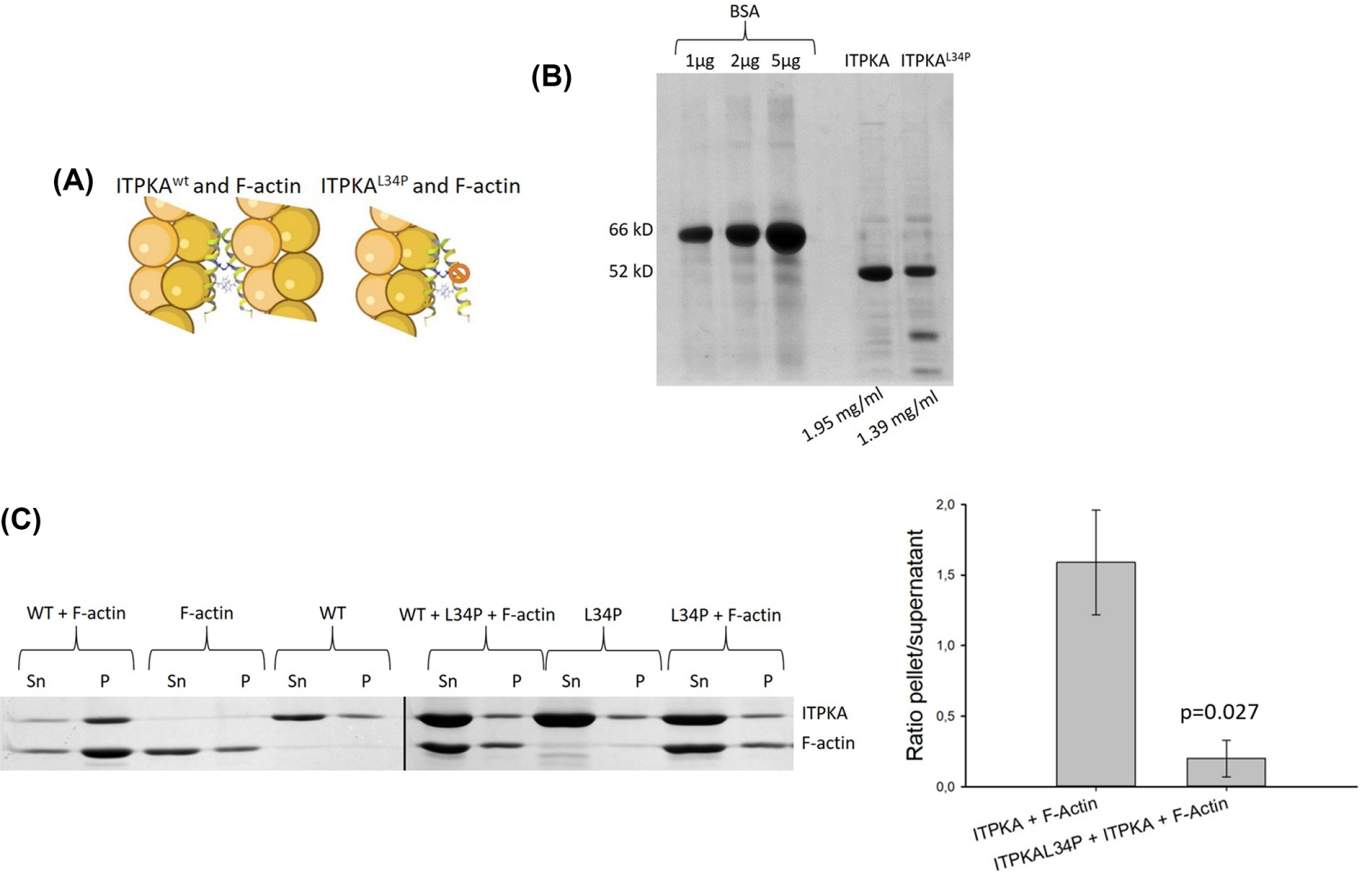
ITPKA^L34P^ is a dominant negative mutant of actin bundling activity of ITPKA (**A**) Cartoon of the effect of ITPKA^L34P^ on actin bundling activity of ITPKA^wt^, created with BioRender. ITPKA^L34P^ is an F-actin binding deficient mutant forming heterodimers with ITPKA^wt^. In this dimer only the wt but not the mutant protein can bind to F-actin and therefore actin filaments are not bundled. (**B**) Purification of ITPKA^wt^ and ITPKA^L34P^. The proteins were recombinantly expressed in bacteria as GFP-His-fusion proteins, enriched by nickel chelate columns, the tags were cleaved with TEV protease and the proteins were finally purified by inverse nickel chelate and size exclusion chromatography. Shown is one representative SDS page using BSA as concentration standard. (**C**) Actin bundling activity of ITPKA in absence and presence of ITPKA^L34P^. About 1 µM F-actin and 0.5 µM ITPKA together with the 10-fold access of ITPKA^L34P^ were pulled down and supernatant (Sn) and pellet (P) were analyzed by SDS page. The right panel shows the ratio from the band intensity of pellet/supernatant from F-actin incubated with ITPKA^wt^ or ITPKA^wt^+ ITPKA^L34P^ including the respective controls (F-actin and the ITPKA proteins alone). Shown are mean values of three independent experiments ± SD.

We speculated that a mutant with defective actin binding activity (ITPKA^L34P^) may inhibit ITPKA’s actin bundling activity by forming heterodimers with wild-type ITPKA (ITPKA^wt^), preventing the binding of two actin filaments (see cartoon in Figure 2A). To validate this idea, ITPKA^wt^ and ITPKA^L34P^ were expressed in bacteria and purified (Figure 2B). Thereafter, an F-actin bundling pull-down assay was performed, employing ITPKA^wt^ and ITPKA^L34P^ in a ratio of 1 to 10, as well as control approaches. We found that ITPKA^wt^ bundled approximately 60% of F-actin and as expected, ITPKA^L34P^ did not show any actin bundling activity. Moreover, ITPKA^L34P^ prevented actin bundling activity of ITPKA^wt^ (Figure 2C), confirming our idea that ITPKA^L34P^ is a negative dominant mutant.

### Repression of ITPKA’s actin bundling activity decreases actin dynamics and migration

**Figure 3:**
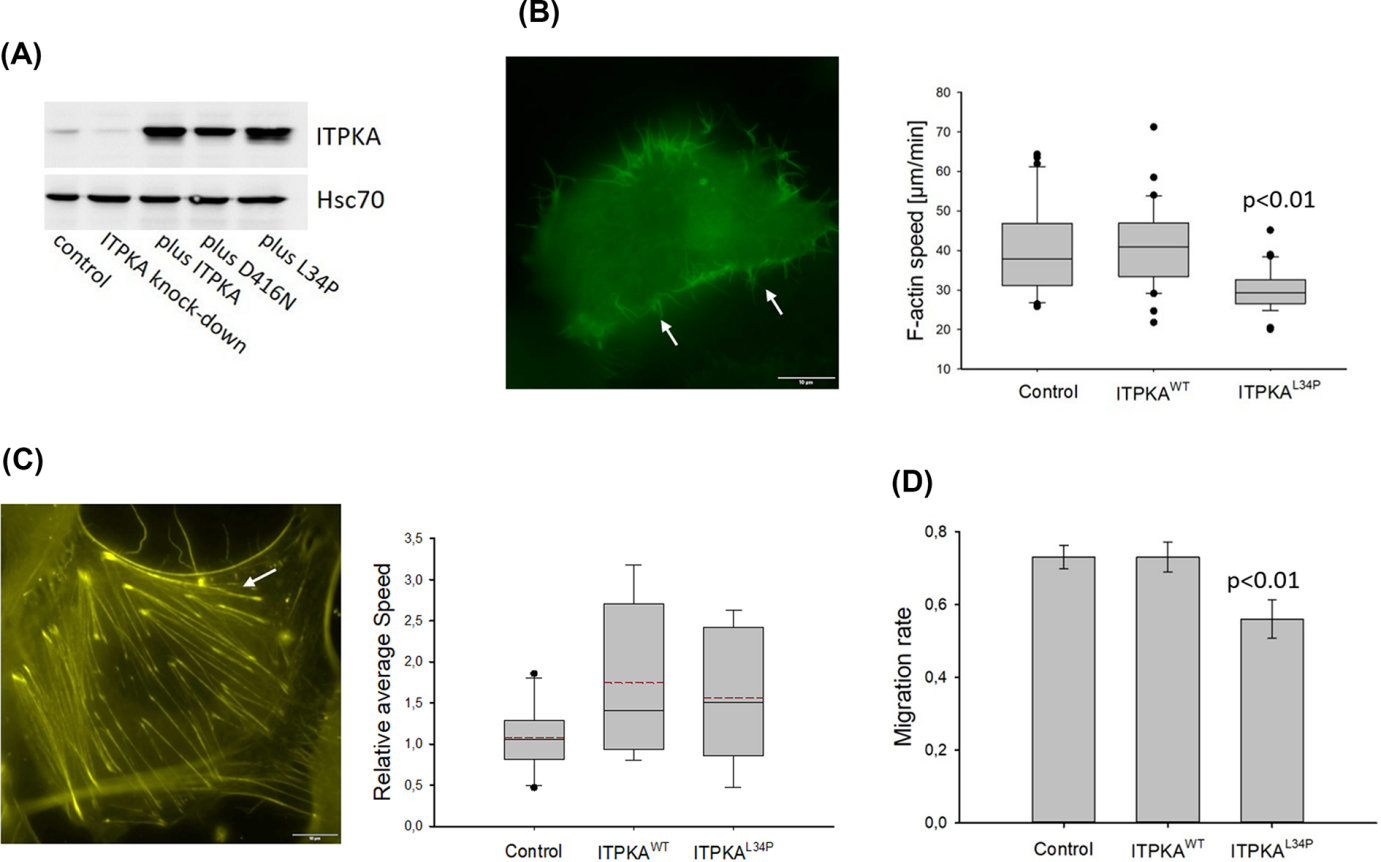
Inhibition of endogenous actin bundling activity reduces migration and motility of filopodia (**A**) ITPKA^wt^, ITPKA^D416N^, or ITPKA^L34P^ were stably overexpressed in H1299 cells using a lentiviral approach, cells transduced with empty Lego vector served as control. Shown is one representative Western-blot, of cell lysates derived from control cells, from ITPKA knock-down cells (as internal control), from control cells overexpressing wtITPKA (plus ITPKA), or a kinase dead mutant (plus D416N, see also Figure 5A), or a dominant negative mutant against the actin bundling activity of endogenous ITPKA (plus L34P). Hsc70 served as loading control. Stable ITPKA expression was controlled monthly. (**B**) Cells were transfected with LifeAct to label F-actin, and motility of filopodia was analyzed by life cell imaging. Right panel shows box plots from 30 independently measured cells. (**C**) Cells were treated with SiR-actin (left panel) and dynamics of actin-myosin fibers were analyzed by kymographs. Right panel shows mean values ± SD of ten independently measured cells. (**D**) Wound healing migration was analyzed by a scratch assay for 16 h and the area closed by the cells was calculated (1 = complete closure). Shown are mean values ± SD from three independent experiments.

To selectively inhibit the actin bundling activity of ITPKA in cells, ITPKA^L34P^ as well as ITPKA^wt^ as control were stably overexpressed in H1299 cells using a lentiviral approach. In Figure 3A and Supplementary Figure S1, the band intensities of ITPKA from protein lysates derived from control cells, from ITPKA knock-down cells, from cells overexpressing ITPKA (plus ITPKA), a kinase dead mutant (plus D416N), or the negative dominant mutant L34P are shown. The results obtained with the cells overexpressing ITPKA^D416N^ are depicted in Figure 5A.

In order to analyze the effect of ITPKA^L34P^ on actin dynamics in cellular protrusion, GFP-LifeAct was transiently expressed in ITPKA manipulated cells and actin dynamics in filopodia were assessed by life cell imaging (Figure 3B). As shown in Figure 3B, actin dynamics were not affected by overexpression of ITPKA^wt^ but were significantly reduced by overexpression of ITPKA^L34P^ (by 25%). We also analyzed actin dynamics of stress fibers in SiR-actin treated H1299 cells but did not find any differences between the cell lines (Figure 3C). Finally, the impact of ITPKA’s actin bundling activity on wound healing migration was analyzed, and it was found that overexpression of ITPKA^L34P^ inhibited migration by 34%, while overexpression of ITPKA^wt^ had no effect (Figure 3D and Supplementary Figure S2).

In summary, inhibition of the actin bundling activity of ITPKA reduced actin dynamics in filopodia and decreased the migratory potential of H1299 lung cancer cells. On the other hand, the actin bundling activity had no effect on actin-myosin motility. From these data we conclude that under basal conditions, ITPKA controls migration of H1299 cells by regulating actin dynamics in cellular protrusions.

### Impact of the actin bundling activity on spheroid growth and on invasion of lung cancer cells

**Figure 4:**
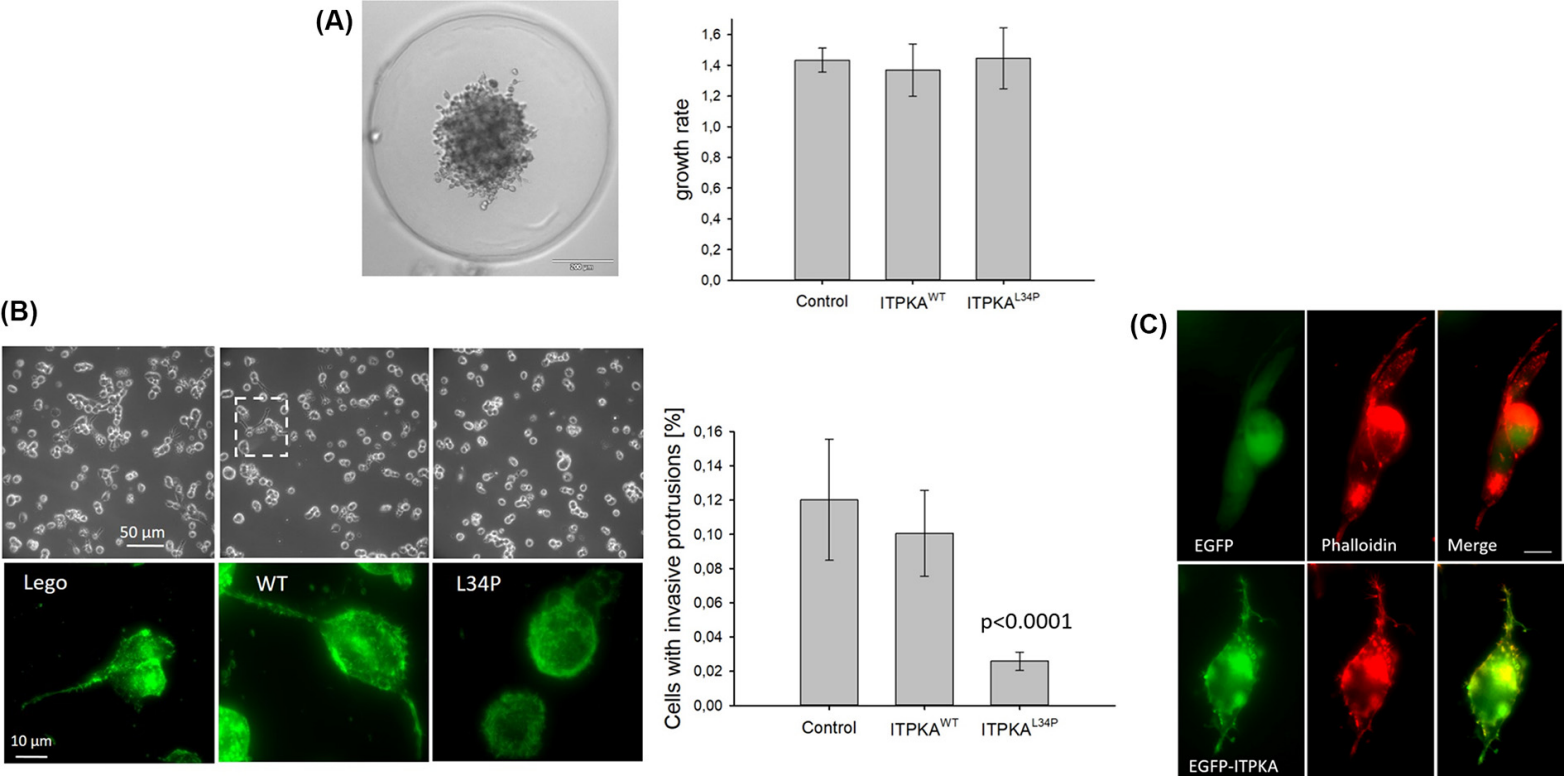
H1299 cell 3D-growth and invasion after inhibition of actin bundling activity of ITPKA (**A**) Cells were grown in molds as spheroids and then fixed with type I collagen. Growth from 35 spheroids were assessed right after type I collagen fixation and after 24 h (right panel). Left panel shows one representative spheroid from control cells, and right panel mean + SD values from three independent experiments. (**B**) Cell suspensions were seeded onto a type I collagen gel, and formation of invasive protrusions were analyzed after 24 h of incubation by counting the cells with invasive protrusions. Left panel shows representative images in the differential interference contrast channel at low magnification (upper panel) and lower panel shows high magnification in the GFP channel; here Alexa-fluor488-conjugated phalloidin stained cells are depicted. Right panel shows the percentage of cells having invasive protrusions as mean ± SD values of three independent experiments. (**C**) Control cells were transfected with a vector encoding for EGFP-ITPKA and after 24 h of incubation the cells were treated as described in (**B**) and imaged by fluorescence microscopy. Shown is one representative image.

In the next step, we analyzed whether the actin bundling activity of ITPKA also affects growth from spheroids and invasion. To assess this, H1299 cells were grown in molds, embedded with collagen I, and the spheroid area was monitored for 24 h (Figure 4A). Invasion was assessed by seeding single cells on to collagen I gels and counting the cells forming invasive protrusions (Figure 4B and Supplementary Figure S3).

After 24 h of incubation, growth from spheroids was not different between the cell lines, but invasion was strongly reduced in H1299 cells overexpressing the dominant negative ITPKA^L34P^ mutant. Approximately 12% of control and 10% ITPKA overexpressing cells formed large invasive protrusions but in cells overexpressing the dominant negative mutant ITPKA^L34P^ only 2% of the cells were able to invade the matrix (Figure 4B, right panel). Thus, inhibition of actin bundling activity inhibited invasion of H1299 cells by 84%, indicating that the actin bundling activity of ITPKA is essential for invasion of H1299 cells into collagen I. In order to show whether ITPKA is specifically located inside the invasive protrusions, control cells were transfected with a vector encoding for EGFP-ITPKA and treated as shown in Figure 4B. However, ITPKA was bound to F-actin all over the cell, including the protrusions. Thus, it does not show a specific localization to invasive protrusions (Figure 4C).

In conclusion, our data reveal that under basal conditions the actin bundling activity of ITPKA accounts for the invasion and migration-promoting effect of ITPKA.

### ITPKA overexpression increases migration in response to cellular ATP stimulation

**Figure 5:**
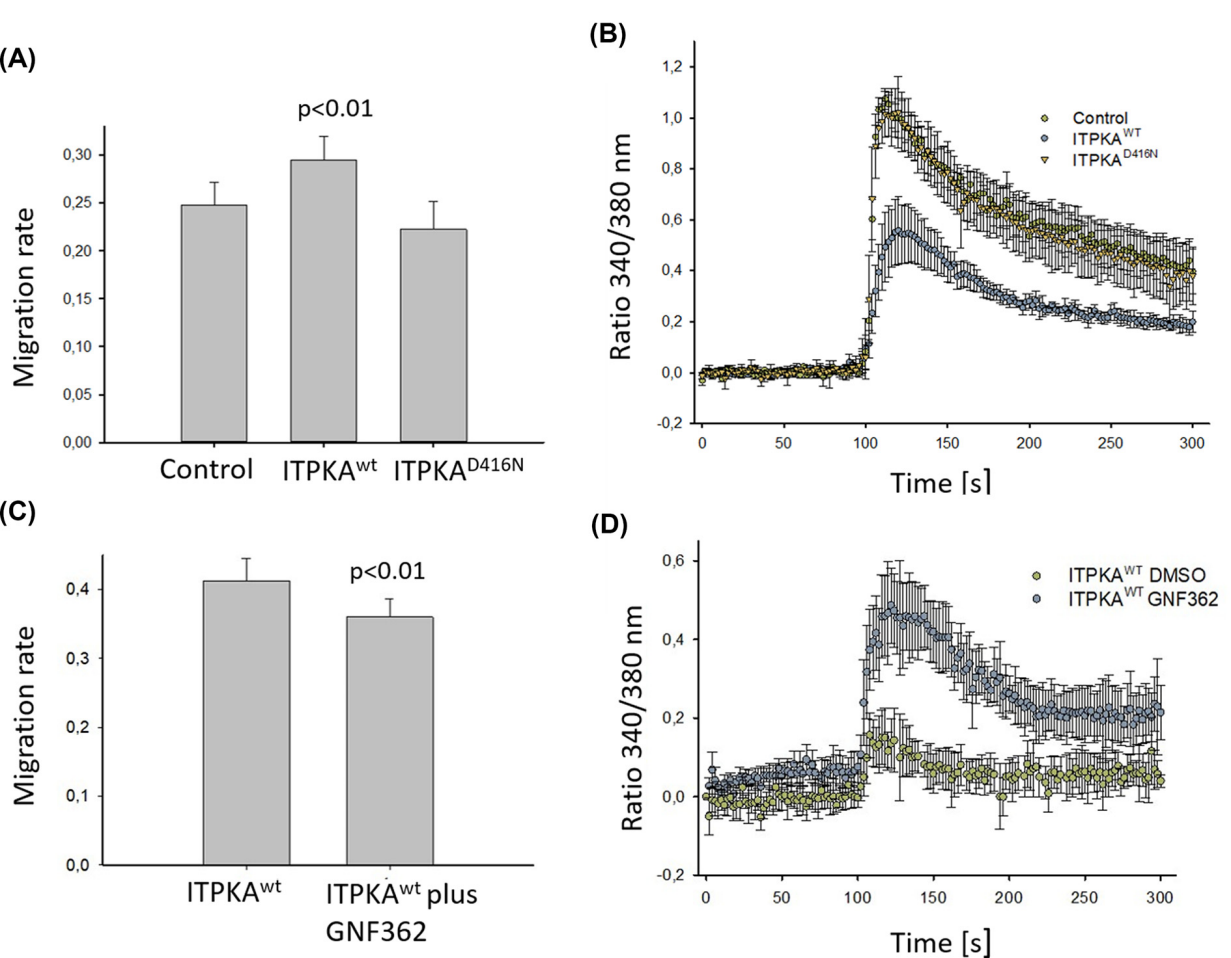
ITPKA overexpression stimulates migration of H1299 cells in presence of ATP, and GNF362 reverses this effect (**A**) Migration was assessed by the scratch assay in presence of ATP for 7.5 h. (**B**) ATP induced calcium signals were measured in Fura-2 loaded cells. (**C**) Cells were treated with 12 µM GNF362 or with DMSO for 16 h, scratched, and after addition of 100 µM ATP wound closure was analyzed for 7.5 h. (**D**) GNF362-treated ITPKA overexpressing H1299 cells were loaded with Fura-2 and ATP-induced calcium signals were measured. Shown are mean ± SD values of three independent experiments.

The experiments conducted to analyze the effect of the actin bundling activity of ITPKA were performed with non-stimulated cells. Under these conditions, the InsP_3_-kinase activity of ITPKA can be neglected because the cellular Ins(1,4,5)P_3_ concentration only increases in response to phospholipase C (PLC) activation [1]. To show if in addition to the actin bundling activity, also the InsP_3_-kinase activity may be involved in ITPKA-stimulated migration, the cells were stimulated with ATP to activate PLC activity. Furthermore, a kinase-deficient ITPKA mutant (ITPKA^D416N^) was overexpressed as control (see Figure 3A). Indeed, under this condition, overexpression of ITPKA slightly but significantly stimulated migration of H1299 cells (by 13%), while the kinase-deficient mutant had no effect (Figure 5A and Supplementary Figure S4). To show if this effect may be associated with altered calcium release from the endoplasmic reticulum ATP-induced calcium signals in Fura-2 loaded cells were measured. The result of this experiment revealed that overexpression of ITPKA^wt^ reduced calcium transients while, as expected, calcium signals in cells overexpressing ITPKA^D416N^ were not different from control cells (Figure 5B).

In order to validate this finding, H1299-ITPKA^wt^ cells were treated with GNF362, a small molecule inhibitor against InsP_3_-kinase activity of ITPKB, which also has been shown to inhibit InsP_3_-kinase activity of ITPKA [19]. Our measurements confirmed inhibition of InsP_3_-kinase activity of ITPKA by GNF362 *in vitro* (IC50 value, 17 nM; competitive to ATP, Figure S5), and analysis of its toxic concentration in H1299 cells revealed that concentrations >12 µM were toxic (Supplementary Figure S6). Therefore, 12 µM GNF362 were applied to assess its effect on migration (Figure 5C and Supplementary Figure S7). In addition, InsP_3_-mediated calcium signals were measured in H1299-ITPKA^wt^ cells in absence or presence of 12 µm GNF362 (Figure 5D). These analyses revealed that GNF362 reversed both, the increased migration rate of H1299 cells overexpressing ITPKA and the reduced calcium signal. Thus, inhibition of endogenous InsP_3_-kinase activity reversed the stimulating effect of ITPKA on H1299 cells.

## Discussion

In former studies, we revealed that a high level of ITPKA increased trans-migration of different types of tumor cells and demonstrated that overexpression of ITPKA in H1299 lung cancer cells significantly increased metastasis in SCID mice [4,9]. In the present study, we confirmed the relevance of ITPKA for metastasis by down-regulating endogenous ITPKA in H1299 cells, and thus validated that both up- and down-regulation of ITPKA control dissemination of lung cancer cells *in vivo*. In our previous study, we already revealed that under stimulating conditions, the InsP_3_-kinase activity contributes to ITPKA-facilitated trans-migration [4]. However, the impact of the actin bundling activity in the process has not been investigated yet.

Therefore, in the present study, we analyzed this issue in detail by assessing migration and invasion, two essential steps in the metastatic cascade [17]. For this purpose, a dominant negative ITPKA mutant (ITPKA^L34P^) was overexpressed in H1299 lung cancer cells to inhibit the actin bundling activity of endogenous ITPKA. To confirm the involvement of the InsP_3_-kinase activity in ITPKA-stimulated migration, we tested the effect of this activity on cellular migration in presence of ATP by overexpressing ITPKA or a catalytic inactive mutant (ITPKA^D416N^).

Our data show that under basal, non-stimulating conditions, overexpression of ITPKA did not further stimulate migration and invasion of H1299 cells. However, inhibition of endogenous actin bundling activity by ITPKA^L34P^ inhibited migration by 34%, and reduced filopodia motility. Since filopodia are required for directed migration by sensing the environment for chemical clues, we strongly assume that the actin bundling activity of ITPKA controls migration by regulating filopodia dynamics. Recently, it has been shown that the F-actin bundles, forming the core inside filopodia, perform a ‘twisting and spinning motion’ [20], and the actin bundling activity of ITPKA forms flexible rotatable and stretchable F-actin cross-links [21]. Therefore, it is very likely that the actin bundling activity of ITPKA is required to form flexible F-actin networks enabling dynamic filopodia movements, essential to sense the environment. The same may be true for the formation of invasive protrusions (see Figure 4B and Supplementary Figure S3), requiring a high flexibility to invade the substrate [22], and whose formation was reduced by 87% after inhibiting ITPKA’s actin bundling activity. The flexible nature of the F-actin network induced by the actin bundling activity of ITPKA may also explain why ITPKA does not play a role for motility of stress-fibers, consisting of rigid F-actin bundles contracted by myosin II [23]. In conclusion, our data strongly indicate that the local effect of ITPKA on F-actin dynamics does not result from accumulation of ITPKA in cellular protrusions (Figure 4C [4,6]) but is due to its specific modulation of the F-actin network.

In addition to the strong effect of the actin bundling activity of ITPKA on migration and on invasion, we found that overexpression of ITPKA slightly increased migration after stimulating the cells with ATP. Our finding that overexpression of a kinase deficient ITPKA mutant had no effect on migration validate this finding, and we previously demonstrated that re-expression of an InsP_3_kinase-deficient mutant in ITPKA knock-down H1299 cells did not fully restore reduced trans-migration of the cells [4]. Moreover, the InsP_3_-kinase inhibitor GNF362 [19] was sufficient to reverse the stimulating effect of InsP_3_-kinase-A activity on migration, again confirming the requirement of InsP_3_-kinase activity for ATP-stimulated migration of H1299 cells. We assume that this effect is due to the calcium-calibrating activity of ITPKA because both down-regulation of endogenous ITPKA (data not shown) and up-regulation of ITPKA decreased ATP-induced Ins(1,4,5)P_3_-mediated calcium signals in H1299 cells. However, further experiments are required to precisely elucidate the role of InsP_3_-kinase-A activity in ATP-stimulated migration.

In summary, our data show that in H1299 lung cancer cells mainly the actin bundling activity of ITPKA promotes migration and invasion, which is slightly enhanced by its InsP_3_-kinase activity after cellular stimulation with ATP. GNF362 had been identified as very potent, inhibitor against InsP_3_-kinase activity *in vitro* and *in vivo* (see [19] and Supplementary Figure S1), thus potentially may serve as a small molecule inhibitor to block InsP_3_-kinase-A activity in cancer cells. However, the compound also inhibits activity of ITPKB, playing an essential role in B-cell maturation and activity of dendritic cells [24,25]. Thus, treatment of cancer patients with GNF362 may decrease antibody- and T-cell dependent tumor cell cytotoxicity [26,27]. Based on this consideration, attempts should be made to develop an isoform-specific inhibitor that only blocks activity of InsP_3_-kinase-A. Moreover, there are no compounds available inhibiting the actin bundling activity of ITPKA, which is the main migration/invasion-driving activity of ITPKA. Thus, it would be of high interest to develop tools inhibiting this ITPKA activity.

## Methods

### Chemicals, antibodies, proteins, and vector

The anti-ITPKA antibody was purchased from Santa Cruz Biotechnology (sc-11206)), the vector encoding for LifeAct from Addgene (mEGFP-LifeAct-7, Addgene, #54610). SiR actin was from Spirochrome (CY-SC001), Collagen I from Roche (11179179001), Fura-2 from AAT Bioquest (21020), Ins(1,4,5)P_3_ from Buchem B. V. (D-1,4,5 IP3-Na-10 mg), and ATP from Carbosynth (NA00135).

### Cell culture

NCI-H1299 (H1299) cells were kindly provided by Cagatay Günes (Hamburg, Germany), for characteristics of these cells see American Type Culture Collection (ATCC, Rockville, U.S.A.). The cells were grown in Dulbecco’s Modified Eagle’s Medium (DMEM) supplemented with 10% (v/v) fetal calf serum (FCS), 4 mM L-glutamine, 100 μg/ml streptomycin, and 100 U/ml penicillin.

### Analysis of H1299 lung cancer cell metastasis in SCID mice

All animal experiments took place at the central mouse facility of UKE, Germany. A total of 1 × 10^6^ H1299 control and shRNA cells (see [4]) suspended in FCS-free DMEM were subcutaneously injected into the neck of BALB/c severe combined immunodeficient SCID mice (*n* = 8–10). Under this conditions, the tumor cells disseminate to the lungs, thus form distant metastasis [4,28]. After the primary tumors had a size of about one diameter, the mice were anesthetised by ketamine/xylazine (100 mg/kg), and killed by cervical dislocation. The lungs were dissected, fixed in formalin, sliced in 5 µM sections and every tenth section was stained with haematoxylin and eosin. In order to evaluate the presence of spontaneous lung metastases, the stained sections of each lung were examined at a 1 × 200 magnification. Irrespective of the size, all distant lung metastases found in one section were counted, see also [4]. Representative micrometastasis are shown in Figure 1, right panel.

This experiment was approved from the Behörde für Gesundheit und Verbraucherschutz, and all aspects of the animal experiments were conducted in accordance with the approved protocol (70/13).

### Recombinant expression and purification of ITPKA

ITPKA was expressed in bacteria as GFP-His-fusion protein, enriched by nickel-chelate and size exclusion chromatography, cleaved by Etch Virus nuclear-inclusion-a endopeptidase (TEV) and purified again by inverse nickel-chelate chromatography and size exclusion chromatography as described [29].

### Western blotting

For the preparation of lysates, cells were washed with phosphate buffered saline (PBS) and harvested in MPER buffer (Promega, Mannheim, Germany) supplemented with protease inhibitor mix (Roche, Alameda, CA, U.S.A.). This suspension was frozen at −80°C, thawed, and then centrifuged (15 min, 15000 × ***g***, 4°C). Protein concentrations of the supernatants were determined using the BCA Protein Assay Kit (Pierce, Rockford, IL, U.S.A.), according to the manufacturer’s instructions. The remaining supernatant was immediately added to Laemmli sample buffer (20 mM Tris-HCl, 2% SDS, 25% glycerol, 0.3% mercaptoethanol, 0,032% bromophenol blue). About 20 µg of protein were analyzed by Western blotting using a standard protocol.

### Actin bundling assay

Actin was prepared from fresh chicken breast as described [30] and stored in G-buffer (10 mM Tris-HCl, pH 7.5, 0.2 mM CaCl_2_). In order to produce F-actin, G-actin was incubated in F-actin buffer (20 mM Tris-HCl, pH 7.5, 100 mM KCl, 2 mM MgCl_2_, 1 mM EGTA, 1 mM DTT, 0.5 mM ATP) in presence or absence of ITPKA proteins, incubated for 20 min at room temperature (RT) and centrifuged at 10,000 ***g*** for 20 min at 4°C. To analyze the concentration of F-actin bundles and F-actin binding proteins in the pellet, supernatants and pellets were analyzed by Coomassie-stained SDS-PAGEs and band intensities were analyzed by ImageJ.

### Stable ITPKA expression of ITPKA and ITPKA mutants in H1299 cells

For overexpression of ITPKA and mutants in H1299 cells, the cDNAs of wt and the ITPKA mutants L34P and D416N were cloned into the lentiviral vector LeGo-iB2-Neo+ which was a friendly gift from Kristoffer Riecken. Virus production in HEK293-T cells and infection of H1299 cells was performed as described [31]. Control H1299 cells were transduced with the LeGo-iB2-Neo+ only.

### Transient transfections

A total of 2.5 × 10^4^ cells were seeded in chamber slides (Ibid). After 16 h of incubation, the cells were transfected with EGFP-ITPKA [4] or pEGFP-C1 LifeAct EGFP (Addgene 58470) using the K2® Transfection Reagent from Biontex according to the manufacturer’s instructions. After further incubation for 24 h, the cells were optionally fixed with 4% paraformaldehyde/4% sucrose, stained with Alexa568-coupled phalloidin, and analyzed by fluorescence microscopy using the IXplore Live microscope imaging system from Olympus.

### Analysis of ATP-induced cellular calcium signals

H1299 cells were suspended, and the Ca^2+^ indicator Fura 2/AM (Calbiochem, EMD Biosciences Inc., San Diego, U.S.A.) was added to the suspension at a final concentration of 4 µM. After 30 min (37°C) the cells were washed twice and medium was replaced with buffer (140 mM NaCl, 5 mM KCl, 1 mM MgSO_4_, 1 mM CaCl_2_, 1 mM Na_2_HPO_4_, 5.5 mM glucose and 20 mM Hepes, pH 7.4). Fura-2 signals were measured at an excitation of 340 and 380 nm and at an emission at 495 nm in an HITACHII F270 fluorimeter, and after assessing the baseline, Ins(1,4,5,)P_3_-dependent Ca^2+^ release from the ER was induced by adding 100 µM ATP. The Ca^2+^ signals were expressed as a ratio 340/380.

### Analysis of actin dynamics in filopodia

About 2.5 × 10^4^ cells were seeded in chamber slides (Ibidi). After 24 h of incubation, the cells were transfected with pEGFP-C1 LifeAct-EGFP (Addgene 58470) using the K2® Transfection Reagent from Biontex according to the manufacturer’s instructions. After incubation for 24 h, time series were taken every 50 s for 17 frames by fluorescence microscopy at 100-fold magnification using the IXplore Live microscope imaging system from Olympus. Three filopodia per cell were measured by analysing its velocity (distance/time; µm/sec) using the Fiji software.

### Measurement of actin dynamics in stress fibers

About 2.5 × 10^4^ cells were seeded in chamber slides (Ibidi). After 24 h of incubation cells were labelled with 200 nM SiR-actin (spirochrome) and 10 µM verapamil for 5 h. Time series images were taken for 1 h every 3 min by fluorescence microscopy at 100-fold magnification using the IXplore Live microscopy system from Olympus. Time series images were then opened with Fiji and a straight line was drawn through one cell. Then, a Kymograph was rendered using the Fiji Kymograph tool, and the Velocity Measurement Tool Macro (Volker Baecker, INSERM, Montpellier, RIO Imaging; J. Rietdorf, FMI Basel; A. Seitz, EMBL Heidelberg). The average speed of every stress fiber was measured, and an average stress fiber speed was calculated for each cell.

### Wound-healing migration

Cells were grown in 6 well plates containing DMEM/10% FCS to approximately 90% confluence and a wound was made by using a yellow pipette tip (0 h). After washing with PBS twice, the cells were grown for 7.5 or 16 h in DMEM/10% FCS, and migration into the wound was monitored by light microscopy at 4-fold magnification (see Supplementary Figure S2). The area of the wound at 0 h and 7.5 or 16 h was determined by using ImageJ free hand setting, and migration was expressed as percent of cells migrating into the wound, here complete wound closure was set to 100%.

### Single cell invasion assay and fluorescent staining of F-actin

Type I collagen gels were prepared to a final concentration of 1 mg/ml and an invasion assay was performed as described [32]. In brief, collagen was spread onto wells of a 6-well plate and the collagen was incubated for 1 h in a humidified atmosphere of 5% CO_2_ at 37°C. Gels were seeded with 10^5^ cells and incubated for 24 h. Sixteen fields of view were automatically captured at fixed points at 20-fold magnification by light microscopy and cells with invasive protrusions were counted (see representative images in Supplementary Figure S3). Invasion rate was expressed as percentage of invasive cells relative to all cells counted. To stain F-actin, gels were cut into small pieces and cells were fixed with 4% paraformaldehyde/4% sucrose and permeabilized with 0.5% Triton X-100 in PBS for 15 min. Thereafter, the gels were blocked with 2% BSA/1% glycine in PBS for 30 min and stained with Alexa488-coupled phalloidin for 30 min. Gels were mounted onto a slide and imaged at 100-fold magnification using the IXplore Live microscope imaging system from Olympus.

### Measurement of spheroid growth

Cells were grown in micro-molds (Merck, MicroTissues® 3D Petri Dish® micro-mold spheroid kit) as spheroids and fixed with type I collagen. Growth of the spheroids were assessed right after collagen I treatment and 24 h later by light microscopy at 4-fold magnification using the IXplore Live microscope imaging system from Olympus. The area covered by the spheroids were measured at time point 0 and 24 h, and growth was measured by calculating the ratio of these areas using the Fiji software.

### Determination of IC50-value and inhibitor type

InsP_3_-kinase activity of ITPKA was analyzed by a coupled optical assay. For this purpose, 30 nM ITPKA was mixed with reaction buffer (20 mM Hepes, pH 7.5; 5 mM MgCl_2_; 30 mM KCl_2_; 1 mM DTT; 1mM phosphoenolpyruvate; 0.2 mM NADH; 1000 U/ml pyruvate kinase and lactate dehydrogenase; 50 µM ATP) in an end volume of 300 µl, and incubated for 10 min at 30°C in. The solution was transferred to a 96 well plates, and the baseline was measured at 365 nm in a Tecan reader Infinite 200. The reaction was started after adding 15 µM Ins(1,4,5)P_3_. For determination of ATP competition, the reaction buffer contained 15 µM Ins(1,4,5)P_3_ and the reaction was started with different ATP concentrations. GNF362 was dissolved in DMSO.

### Statistical analysis

Student’s *t*-test was applied for comparison between two groups using SigmaPlot14.

## Supplementary Material

Supplementary Figures S1-S7

## Data Availability

All relevant data are included in this article or in Supplementary Material.

## Abbreviations

F-actin: filamentous actin
GFP: green fluorescent protein
INPP4: 5′phosphatase
ITPKA: Ins(1,4,5)P_3_-kinase-A
PLC: phospholipase C
