# Supplementary material for: The actin bundling activity of ITPKA mainly accounts for its migration-promoting effect in lung cancer cells
Source: Biosci Rep. 2023 Feb 9;43(2):BSR20222150. doi: 10.1042/BSR20222150 (PMC9912108; doi:10.1042/BSR20222150)

Figure Legends Supplemental Figures

Figure S1. Original Western blots of Figure 3A. To demonstrate the high quality of the cropped Western blots shown in Figure 3A, an uncropped Western blot is shown. Upper panel: Western blot treated with anti-ITPKA antibody, lower panel: The same blot treated with an antibody against HSC70.

Figure S2. Determination of the migration rate in control cells and cells overexpressing ITPKA^{WT} or ITPKA^{L34P}. Representative images of a scratch assay demonstrate the determination of the migratory potential. After the scratch was made, a certain area was marked on the bottom of the Petri-dish, to ensure that the same scratch area is analysed after 0 h and after 16 h. The area between these bold black lines was marked by freehand (blue line), and calculated by Image J. To calculate the migration rate, the area after 0 h was subtracted from the area after 16 h (area 0 h – 16h). For each experiment, three scratches/cell line were performed and mean values were calculated. Finally, the mean values of three independent experiments ± SD were calculated.

Figure S3. Determination of the invasion rate in control cells and cells overexpressing ITPKA^{WT} or ITPKA^{L34P}. Shown are 4 representative images per cell line, invasive cells are marked by white arrows, and one area (middle panel) with invasive cells is enlarged. 16 images were automatically generated, cell number was determined and those cells having invasive protrusions were counted. Finally, the mean values of three independent experiments ± SD were calculated.

Figure S4. Scratch assay in presence of 100 µM ATP. Representative images from scratch assays after 0 h and after 7.5 h are shown. The migratory potential of the cells was determined as described in Figure Legend 2.

Figure S5. Validation of GNF362 on InsP$_3$Kinase-A inhibition. A and B. InsP$_3$Kinase activity of 30 nM ITPKA was analyzed by the coupled optical assay (see methods), in presence or absence of different GNF362 concentrations (A) or at 25 nM GNF362 and different ATP concentrations (B, C).

Figure S6. Determination of the toxic concentration of GNF362. (A) Viability of H1299 control cells incubated in absence (0 µM) or in presence of different GNF362 concentrations was measured by the MTT assay over a time course of 48 h. **(B)** Viability of control H1299 cells and of two H1299 cell lines with ITPKA knock down (sh1 and sh2) was measured by the MTT assay over a time course of 48 h. Shown are mean values ± SD of three independent experiments.

Figure S7. Scratch assay in presence of 100 mM ATP and 12 µM GNF362. Representative images from scratch assays after 0 h and after 7.5 h are shown. The migratory potential of the cells was determined as described in Figure Legend 2.

Figure S1

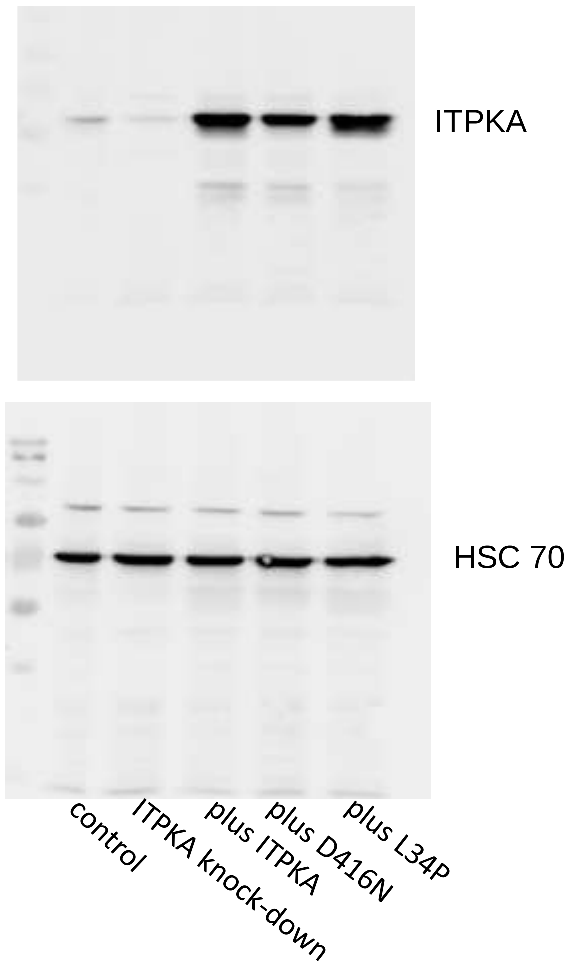
ITPKA
HSC 70
control
ITPKA knock-down
plus ITPKA
plus D416N
plus L34P

Figure S2

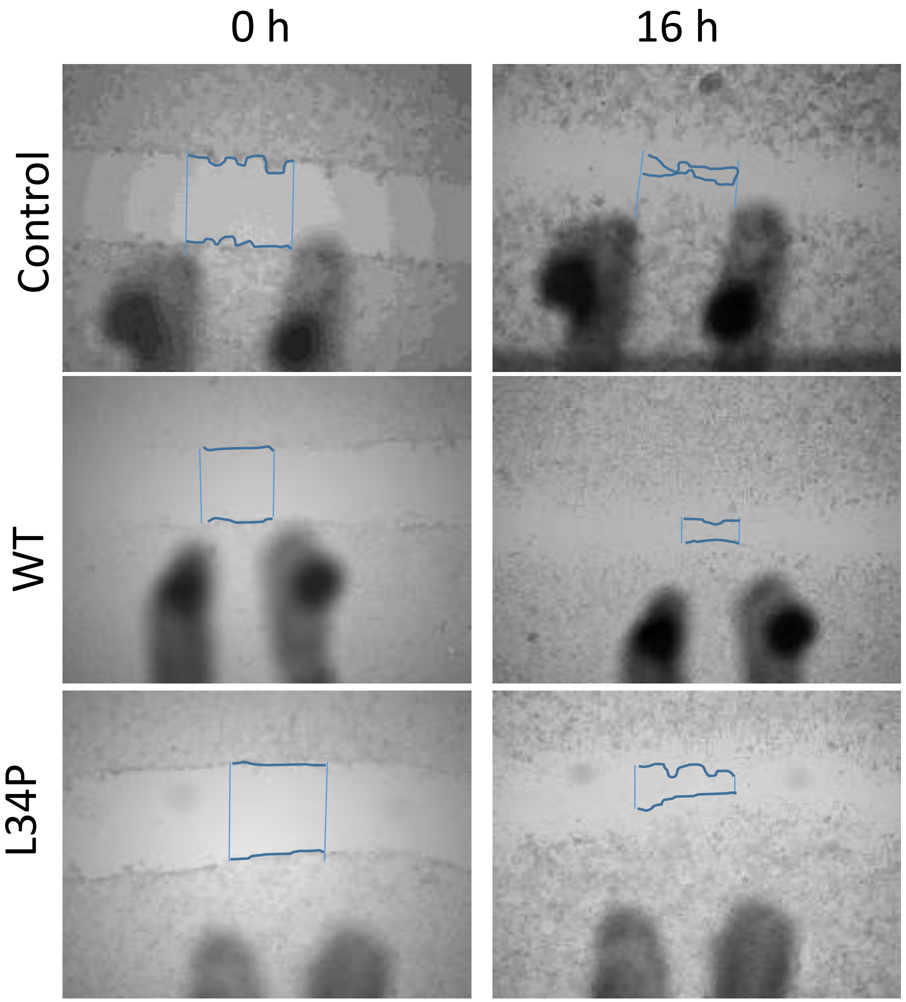

Figure S3

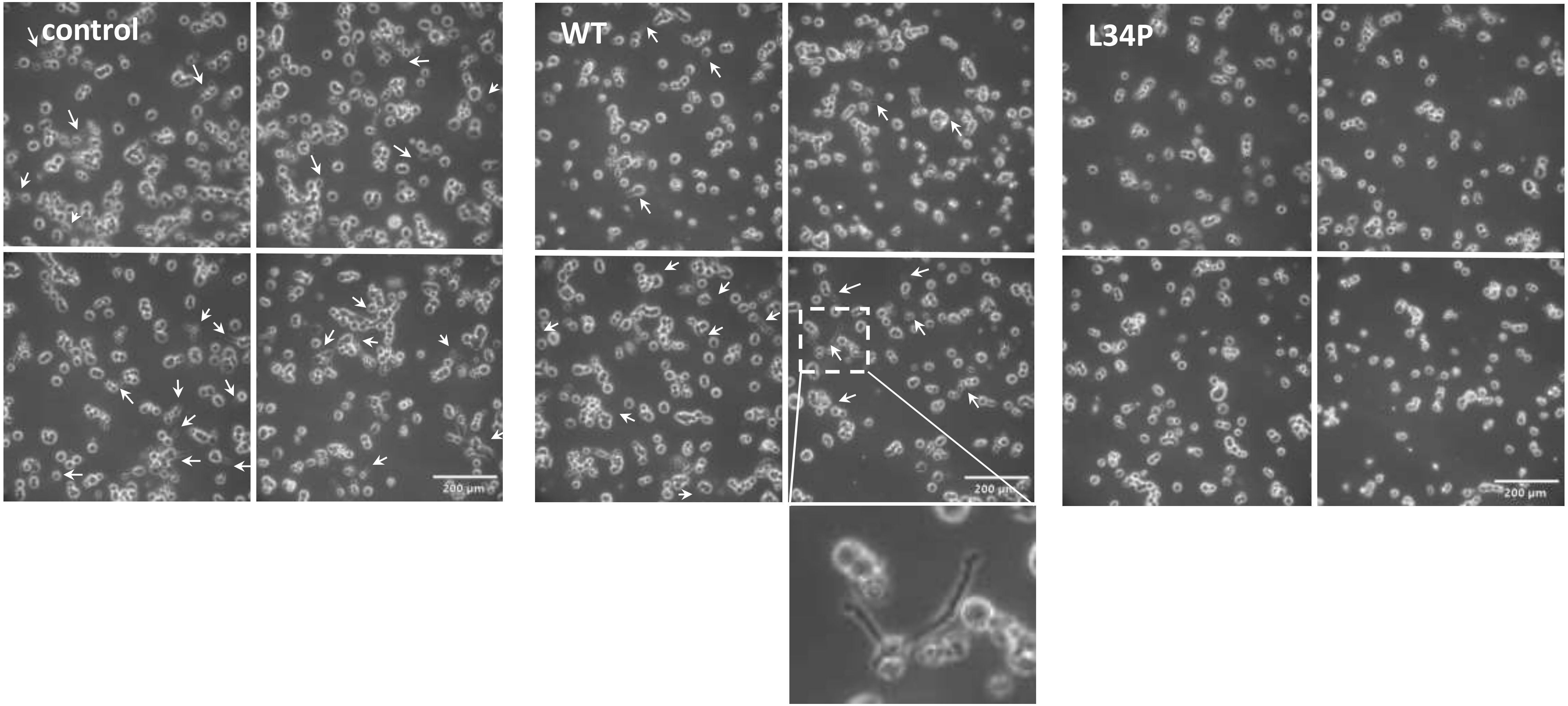

Figure S4

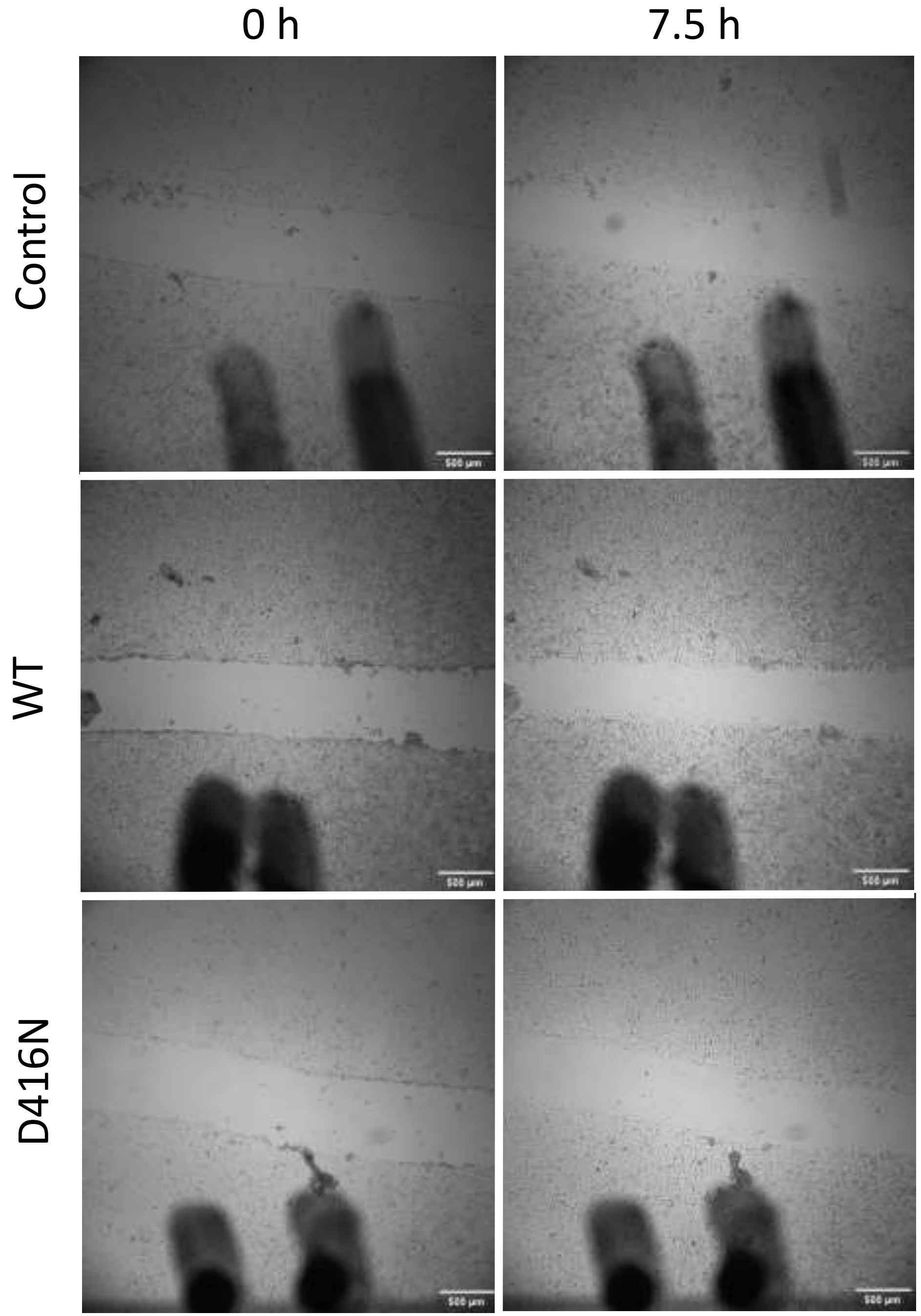

Figure S5

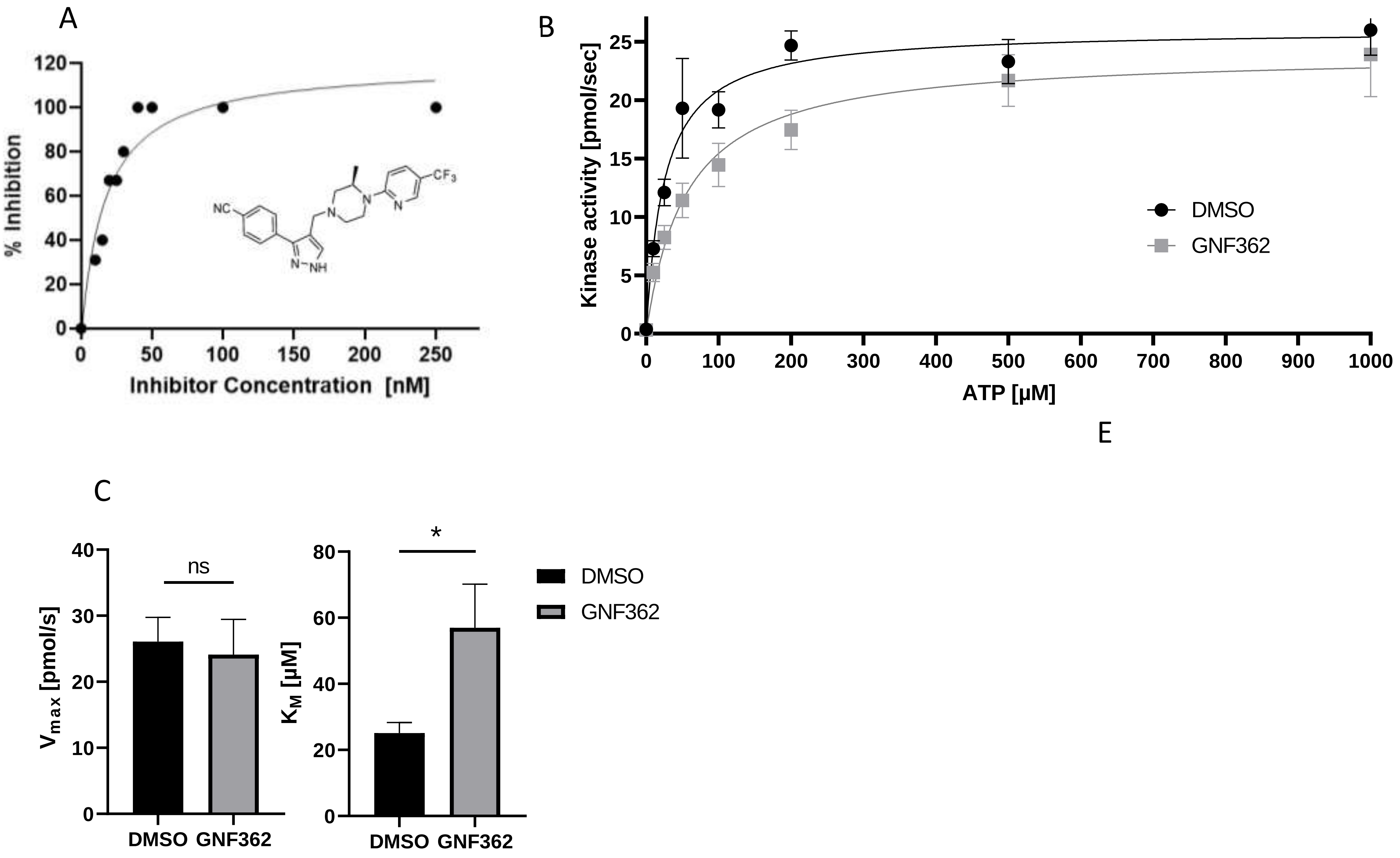
A
% Inhibition
Inhibitor Concentration [nM]
NC
CF3
N
N-NH
B
Kinase activity [pmol/sec]
ATP [µM]
DMSO
GNF362
E
C
ns
V_{max} [pmol/s]
DMSO
GNF362
*
K_M [µM]
DMSO
GNF362
DMSO
GNF362

Figure S6

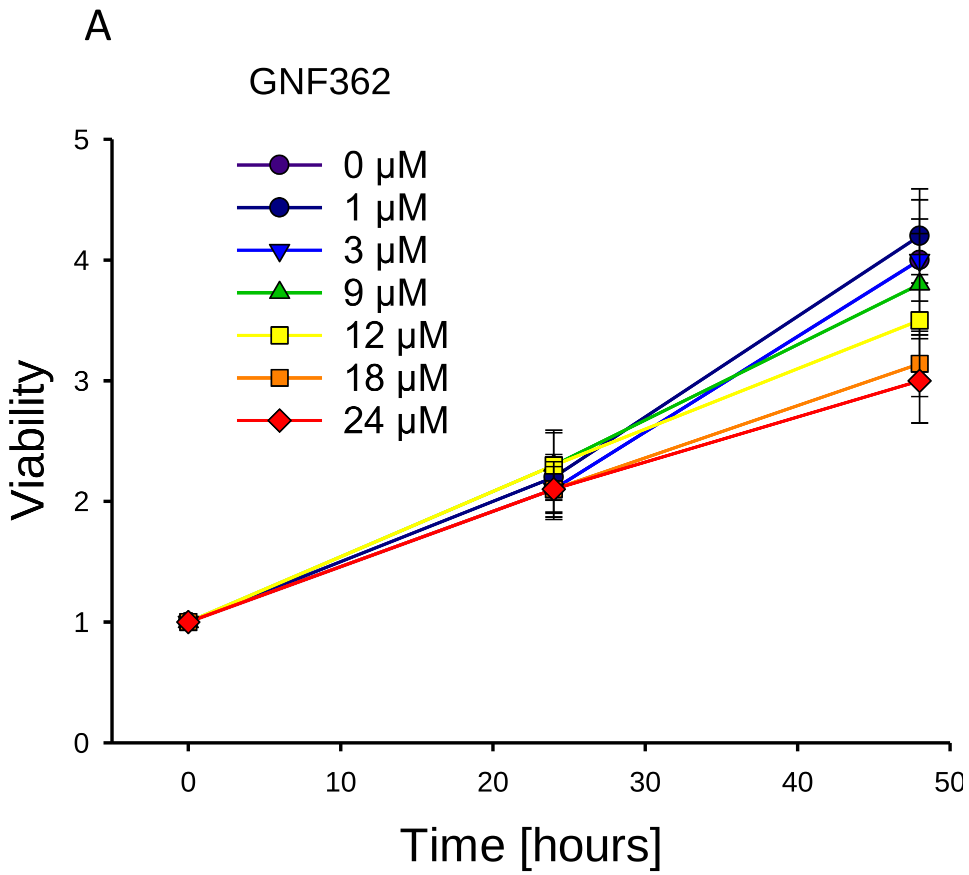
A
GNF362
0 μM
1 μM
3 μM
9 μM
12 μM
18 μM
24 μM
Viability
Time [hours]
0
1
2
3
4
5
0
10
20
30
40
50

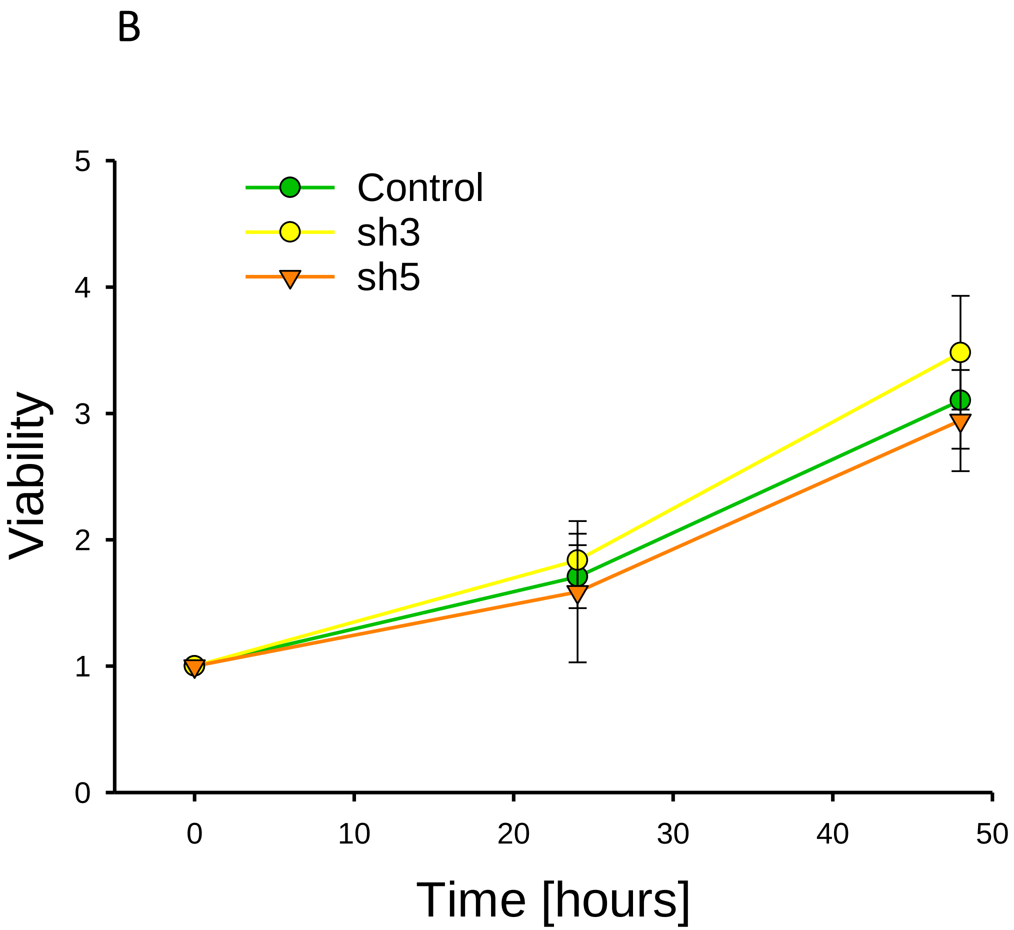
B
Control
sh3
sh5
Viability
Time [hours]
0
1
2
3
4
5
0
10
20
30
40
50

Figure S7

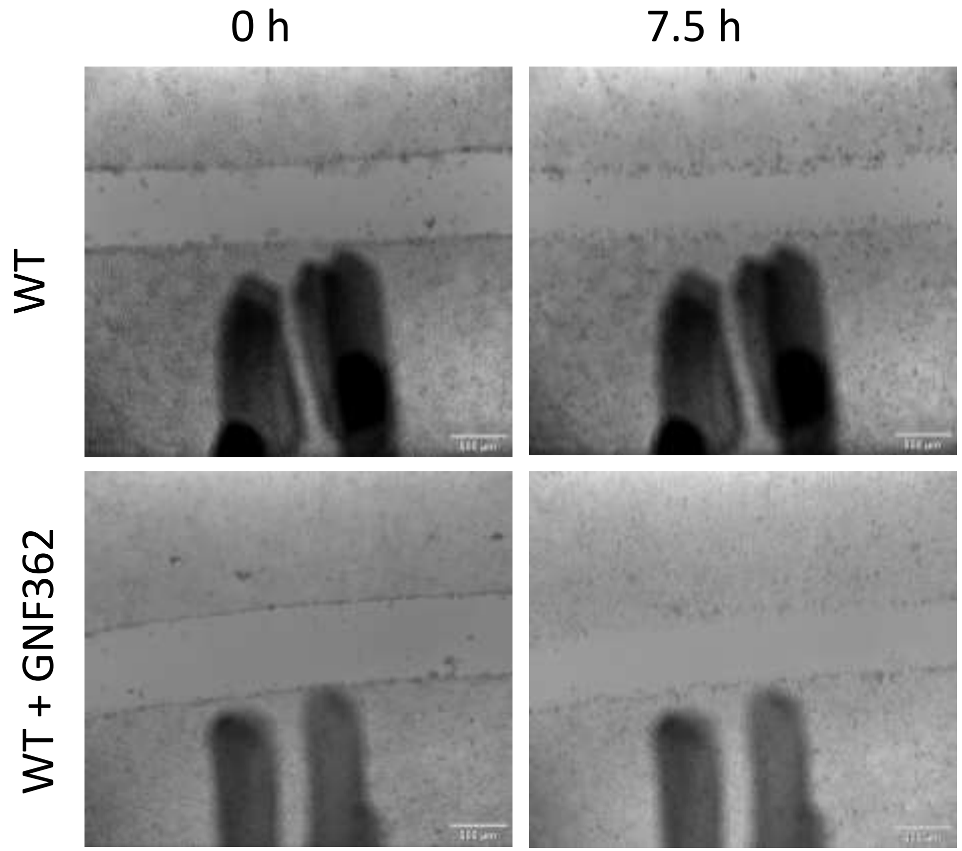

Supplement: Supplementary Figures S1-S7 [file BSR-2022-2150_supp.pdf]
